# A Rare Simultaneous Occurrence of Splenic and Pelvic Cavity Hydatid Cyst

**DOI:** 10.7759/cureus.20827

**Published:** 2021-12-30

**Authors:** Hossein Torabi, Kasra Shirini, Rona Ghaffari

**Affiliations:** 1 Department of General Surgery, Poursina Medical and Educational Center, Guilan University of Medical Sciences, Rasht, IRN; 2 Department of General Surgery, Iran University of Medical Science, Tehran, IRN

**Keywords:** pelvic cavity, echinococcus granulosus larvae, spleen, laparotomy, hydatidosis, hydatid cyst

## Abstract

Hydatid cyst is a significant health-threatening problem that can affect almost all organs, especially the lungs and the liver, but the possibility of its occurrence in organs such as the spleen or pelvic cavity is rare. Thus, simultaneous hydatid cysts in the spleen and pelvic cavity are probably very rare. Nevertheless, since hydatid cysts in different areas can cause various symptoms, it should be considered a significant diagnosis. This article presents a case report of a 21-year-old woman presenting with right lower quadrant abdominal pain mimicking appendicitis but found to have simultaneous hydatid cysts in the spleen and the pelvic cavity.

## Introduction

Hydatid cyst is a serious health problem that is endemic in many regions worldwide, such as the Mediterranean, South Africa, and the Middle East, including Iran. Iran is one of the countries with a high prevalence of hydatid cyst disease, where this problem can be observed more. [1,2]. Approximately 1% of patients admitted to the operation room in Iran are patients with hydatid cysts [3]. Hydatid cyst is a serious infectious disease caused by *Echinococcus granulosus* larvae [4]. It is possible to find hydatid cysts in almost any organ. The main sites are the liver (50%-77%) and the lungs (15%-47%). However, it is less likely to be found in other organs such as the spleen (2%-4%) and kidneys (0.5%-8%). Hydatid cyst in the pelvic cavity could be the primary type, or it could be caused by kidney and spleen hydatic cyst rupture [3,5]. The symptoms and clinical appearances of patients depend on the location of the hydatid cyst. Extrahepatic hydatid cysts almost cause abdominal pain, fever, and anorexia. The most important diagnostic ways are patient’s clinical presentation, serology test, and using diagnostic tools such as ultrasonography, X-ray imaging, and computed tomography (CT) scan. However, serology tests are less reliable because of high false-negative and false-positive results in up to 15% to 20% cases. The main way to treat and eradicate hydatid cyst is surgery [6,7]. Unusual locations can cause various symptoms and lead to misdiagnosis or delay in diagnosis and threat patients' health conditions [8]. Many studies showed hydatid cyst rupturing, and leakage of its content into the body could dramatically increase the risk of septic and anaphylactic shock [9,10]. Therefore, careful examination of the patients' symptoms, performing accurate physical examinations, and having sufficient knowledge about this disease can be beneficial for patients' correct and timely diagnosis and treatment. In this report, a young woman was presented with an unusual presentation of simultaneous splenic hydatid cyst and pelvic area hydatid cyst with a clinical presentation mimicking appendicitis.

## Case presentation

**Figure 1:**
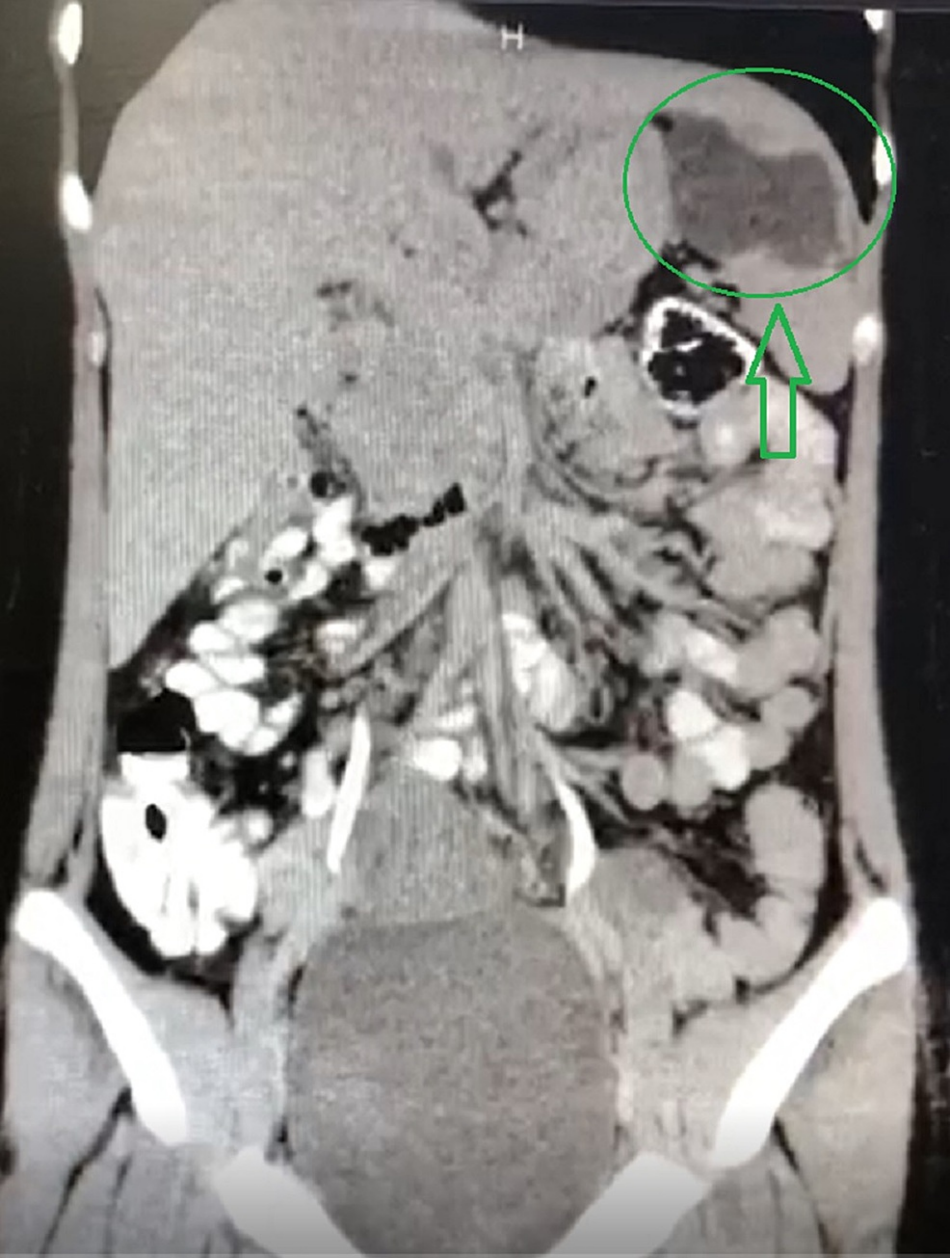
Coronal view of splenic hydatid cyst on abdominal CT scan The green arrow shows the location of the splenic hydatid cyst

**Figure 2:**
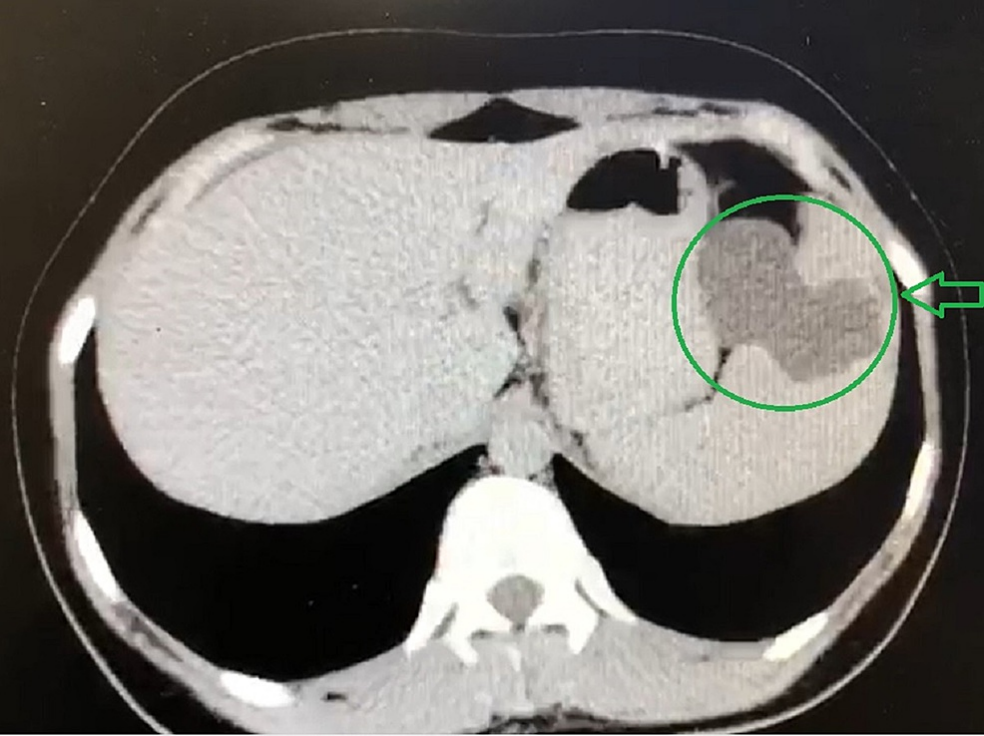
Axial view of the splenic hydatid cyst on abdominal CT scan The green arrow shows the location of the splenic hydatid cyst

A 21-year-old female was presented to the Surgical Department of Poursina Hospital Medical Center, Rasht, Iran, in 2020 with a two-month intermittent right lower quadrant colic pain, nausea after eating, anorexia, urinary frequency, intermittent low-grade fever, and weight loss that was about five kilograms during the last two months. The highest fever recorded by the patients was 38 degrees Celsius, which decreased spontaneously after a few hours without using medication. She claimed that her pains and signs emerged gradually from two months ago, and her pains increased dramatically in the last five days. Our further investigations showed that her past medical history was unrevealing. The physical examination was also benign. She had a sonography report from two weeks before her presentation to the Surgical Department, consistent with appendicitis. Surgeons advised her to undergo another ultrasonography in the hospital for the next step. The ultrasonography report indicated a multilobular heterogeneous cyst, which made us suspect the existence of a hydatid cyst and some little mesenteric lymph nodes in the right lower quadrant. She was asked to undergo an upright chest X-ray and an upright abdominal X-ray for further investigation. In the chest X-ray and abdominal X-ray, no pathological finding was signified. Therefore, she was asked to undergo transvaginal sonography and a CT scan in corneal and axial views, which helped surgeons make better decisions. The transvaginal sonography and CT scan reports confirmed the ultrasound findings and probability of splenic hydatid cyst, as can be seen in Figures 1, 2. However, the reports confirmed that the suspicion of appendicitis was rejected.

**Figure 3:**
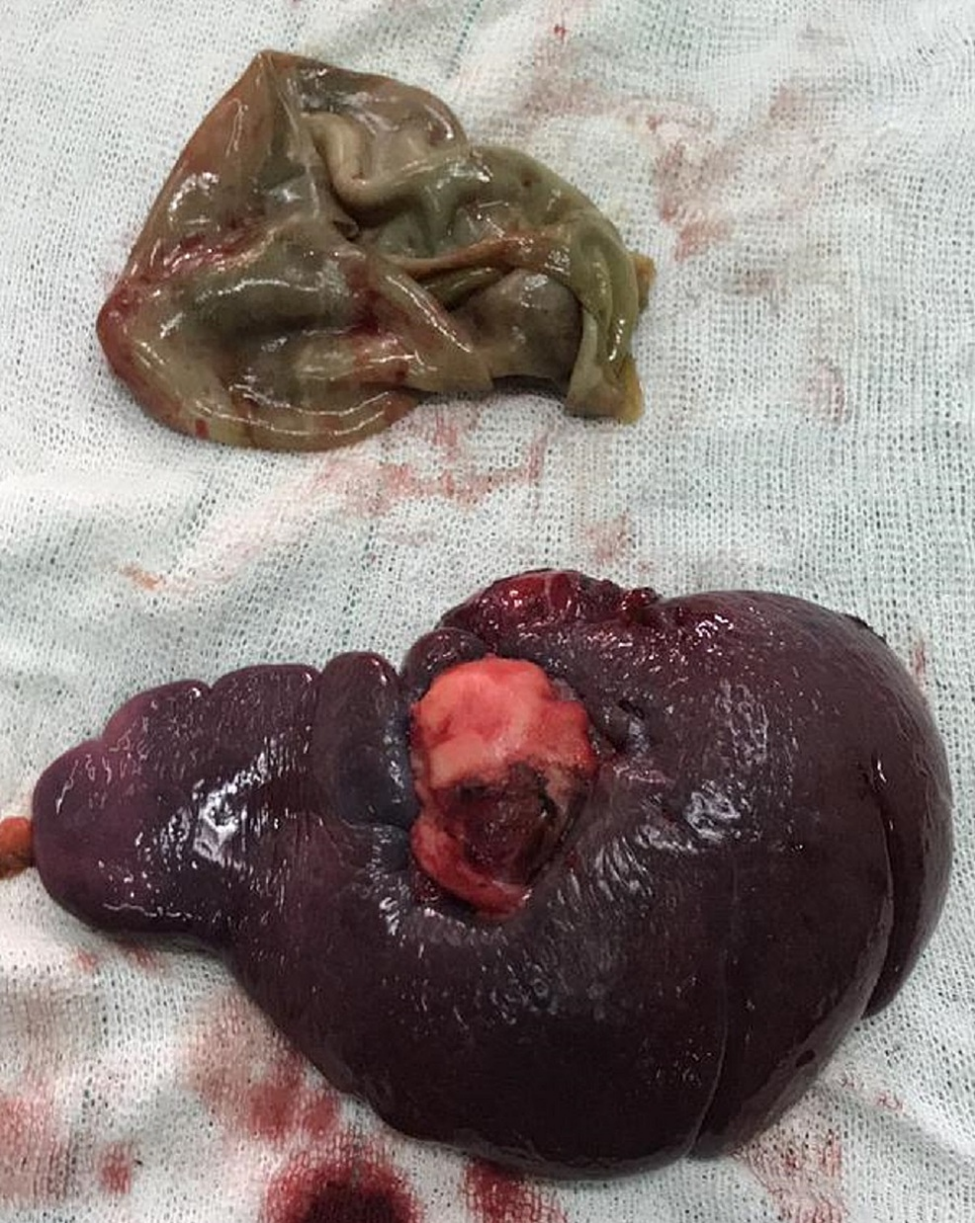
Splenic and pelvic cavity hydatid cysts after excision

The blood tests analyzed presented leukocytosis (white blood cells [WBC] = 24,200 g/dL with a neutrophilia ratio of 90%). In addition, anti-*Echinococcus* antibody immunoglobulin M and immunoglobulin G were positive. Other laboratory studies were within normal limits. Due to suspicion of splenic hydatid cyst and according to the clinical presentations and result of blood tests and imaging reports, the patient underwent laparotomy. A large hydatid cyst measuring 48 x 53 mm was seen in the patient’s spleen during the procedure. Therefore, after the closure of the spleen’s arteries, splenectomy was carefully performed without rupturing the enclosed splenic hydatid cyst. However, a huge and priorly ruptured cyst with completely absorbed contents was observed in the patient’s pelvic cavity. Fortunately, it did not threaten the patient’s health condition, as many studies showed that hydatid cyst rupture and leakage into the body could dramatically increase the risk of septic and anaphylactic shock [9,10]. After removing both cysts, as shown in Figure 3, the pelvic area was washed with hypertonic saline and the abdomen was closed. The patient had a good recovery. On postoperative day 1, her vital signs were within normal limits, and on postoperative day 4, she was discharged. The postoperative histopathological report confirmed that both of them were hydatid cysts.

## Discussion

This article presented a case of simultaneous occurrence of splenic and pelvic cavity’s hydatid cyst, which occurred in a young female who had an unusual presentation of two large hydatid cysts involving the spleen and the pelvic cavity mimicking appendicitis. Hydatidosis is a parasitic zoonosis and health disease that could occur in people who live in farming communities such as the Middle East region, including Iran, by *Echinococcus granulosus* larvae [1,2,11]. This disease could affect different organs of the human body and cause cysts called hydatid cysts [10,11]. The main sites where hydatid cysts could be found are the liver and lungs. However, it could be found in other organs, but hydatid cysts are less likely to be found in the spleen and pelvic cavity than elsewhere [3,11]. Hydatid cysts in the pelvic cavity are usually caused by kidney and spleen hydatic cyst rupturing; however, it could be a primary type. [9]. Hydatid cysts in the pelvic cavity can grow significantly, engross the pelvic area. Hydatid cysts can cause urinary tract symptoms such as urinary frequency or radiational pains such as right lower quadrant pain, as seemed in this case report [12,13]. The main and the most valuable and effective way to diagnose and evaluate hydatid cysts such as splenic hydatid cysts is imaging such as ultrasonography, X-ray imaging, and CT scan [7,11]. Serology tests such as the enzyme-linked immunosorbent assay (ELISA), the indirect hemagglutination test (IHA), the latex agglutination test, and immunoblots could be helpful in diagnosing the disease. However, the results could not be reliable because these tests have high false-positive and false-negative rates [6,7]. There are many surgical techniques to treat splenic and pelvic hydatid cysts. Choosing which surgical technique is more appropriate for the patient depends on the patient’s condition and surgical team, but the open splenectomy for spleen treatment and total excision of hydatid cyst in the pelvic cavity were used in this case [14]. Medications such as mebendazole or albendazole are usually prescribed to patients adjuvantly to prevent recurrences [3]. In this case, the patient was advised to use albendazole to prevent recurrences as much as possible. So far, few cases of hydatid cysts presenting simultaneously in the spleen and other organs and body areas in different people have been reported as case reports [15,16]. Therefore, given the importance of the issue and the importance of paying attention to this disease, we have reported another case in this article.

## Conclusions

Hydatid cyst disease is a significant problem that can be found worldwide, especially in endemic areas such as Iran. Hydatid cyst can cause many symptoms, such as urinary tract symptoms and radiational pains; as stated in this case, the patient presented with right lower quadrant pain and mimicking appendicitis. Therefore, unusual presentations can be misleading and delay diagnosis and treatment with potentially life-threatening complications. Therefore, it is essential to be diagnosed and treated as soon as possible. Thus, it can be concluded that hydatid cyst should be considered a serious differential diagnosis in patients, especially those presenting with abdominal pains in endemic areas.
